# Distinct PINK1‐PRKN mitophagy alterations in different primary tauopathies

**DOI:** 10.1002/alz70861_108879

**Published:** 2025-12-23

**Authors:** Xu Hou, Hannah Hompesch, Savannah J Tiemann, Julian Rubiano, Shunsuke Koga, Taylor Hsuan‐Yu Chen, Melissa E. Murray, Dennis W. Dickson, Fabienne C Fiesel, Wolfdieter Springer

**Affiliations:** ^1^ Mayo Clinic, Jacksonville, FL USA

## Abstract

**Background:**

Mitophagy constitutes a crucial cellular pathway in mitochondrial quality control, in which the enzymatic pair PINK1 and PRKN selectively decorates damaged mitochondria with phosphorylated‐ubiquitin (pS65‐Ub), facilitating their autophagic‐lysosomal removal. We previously demonstrated significant accumulation of pS65‐Ub labeled damaged mitochondria in Lewy body and Alzheimer’s disease autopsy brain. This mitophagy alteration was strongly associated with pre‐tangle tau pathology in neurons. The effects of distinct tau species on mitophagy across different cell types however remain largely unknown. We therefore expanded our analyses to primary tauopathies, exploring how specific tau forms and their aggregation influence mitophagy in selectively vulnerable brain regions and cell types.

**Method:**

This study included 92 control and tauopathy cases with primary age‐related tauopathy (PART), Pick’s disease (PiD), progressive supranuclear palsy (PSP), or corticobasal degeneration (CBD). Immunohistochemistry for the mitophagy marker pS65‐Ub and phospho‐tau was performed in vulnerable brain regions (hippocampus for PART and PiD, primary motor cortex for PSP and CBD). A deep learning‐based image identification model was used to identify and quantify distinct tau inclusions. pS65‐Ub‐positive cells were manually counted and grouped based on signal intensity and morphology. Immunofluorescence co‐staining of pS65‐Ub and phospho‐tau was conducted to study their spatial relationship at single‐cell level.

**Result:**

We observed marked increases in pS65‐Ub‐positive cells in the hippocampus of PART and PiD cases compared to controls. Although less prominent, significant increases were also found in the primary motor cortex of PSP, whereas only minor changes were observed in CBD. Single‐cell analyses revealed that the accumulation of pS65‐Ub was most abundant in hippocampal neurons from PART. Cells with lower signal intensity contained mostly granular pS65‐Ub deposits, while those with higher intensity harbored rather vacuolar inclusions. Across tau aggregate subtypes, pS65‐Ub levels were strongly correlated and often co‐resided with neurofibrillary tangles and Pick bodies. Such correlations were weaker for coiled bodies and tufted astrocytes, and absent for astrocytic plaques.

**Conclusion:**

Our study highlights distinct mitophagy alterations in primary tauopathies that are associated with specific tau inclusions in neurons, oligodendrocytes, and astrocytes. The observed pathological heterogeneity and complex mitophagy disruption underscores the need for future studies to elucidate pathogenic mechanisms and advance targeted therapeutics for each tauopathy.

